# Hypothermic circulatory arrest at 20 ℃ does not deteriorate coagulopathy compared to 28 ℃ in a pig model

**DOI:** 10.1007/s10047-024-01449-9

**Published:** 2024-05-23

**Authors:** Hayato Ise, Kyohei Oyama, Ryohei Ushioda, Aina Hirofuji, Keisuke Kamada, Yuri Yoshida, Payam Akhyari, Hiroyuki Kamiya

**Affiliations:** 1 Department of Cardiac Surgery, Asahikawa Medical University, Midorigaoka-Higashi 2-1-1-1, Asahikawa, Hokkaido 078-8510 Japan; 2 Department of Cardiac Surgery, University Hospital RWTH Aachen, Aachen, Germany; 3 Department of Vascular Surgery, Asahikawa Medical University, Asahikawa, Hokkaido Japan

**Keywords:** Hypothermic circulatory arrest, Cardiopulmonary bypass, Coagulopathy, Bleeding, Animal model

## Abstract

It is believed that a lower temperature setting of hypothermic circulatory arrest (HCA) in thoracic aortic surgery causes coagulopathy, resulting in excessive bleeding. However, experimental studies that eliminate clinical factors are lacking. The objective of this study is to investigate the influence of the temperature setting of HCA on coagulation in a pig model. Ten pigs were divided into the following two groups: moderate temperature at 28 °C (group M, n = 5) or lower temperature at 20 °C (group L, n = 5). Two hours of HCA during a total of 4 h of cardiopulmonary bypass (CPB) were performed. Blood samples were obtained at the beginning (T1) and the end (T2) of the surgery, and coagulation capability was analyzed through standard laboratory tests (SLTs) and rotational thromboelastometry (ROTEM). In SLTs, hemoglobin, fibrinogen, platelet count, prothrombin time, and activated partial thromboplastin time were analyzed. In ROTEM analyses, clotting time and clot formation time of EXTEM, maximum clot firmness (MCF), and maximum clot elasticity (MCE) of EXTEM and FIBTEM were analyzed. Fibrinogen decreased significantly in both groups (group M, p = 0.008; group L, p = 0.0175) at T2, and FIBTEM MCF and MCE also decreased at T2. There were no differences regarding changes in parameters of SLTs and ROTEM between groups. CPB decreases coagulation capacity, contributed by fibrinogen. However, a lower temperature setting of HCA at 20 °C for 2 h did not significantly affect coagulopathy compared to that of HCA at 28 °C after re-warming to 37 °C.

## Introduction

Hypothermic circulatory arrest (HCA) is an essential procedure for distal anastomosis and protects organs from ischemic damage during aortic surgery [1]. Although the outcomes of aortic surgery under HCA with cerebral perfusion today are satisfactory, coagulopathy still remains a main life-threatening complication [2, 3].

In the last two two decades, mild to moderate HCA with a temperature greater than 28 °C is being used more commonly because a lower temperature setting of HCA (deep: 27.9 °C−21.0 °C, profound: < 20.9 °C) has been believed to cause coagulopathy, and surgeons intend to avoid the risk of bleeding [4, 5]. Several recent studies using higher temperature settings of HCA have reported a successful reduction of bleeding and transfusion, without any increase in other complications [6–8]. Conversely, other studies reported that a higher temperature setting of HCA did not reduce bleeding and transfusion [9, 10]. It is still controversial whether a lower temperature setting of HCA deteriorates coagulopathy and increases perioperative bleeding. It is important to investigate the single effect of the temperature setting in HCA. However, in vitro and animal studies with the elimination of clinical factors are lacking.

Our previous study, in which HCA was simulated in vitro, indicated that exposure to hypothermic temperatures did not cause irreversible coagulopathy [11]. Our results made us skeptical about the notion that a lower temperature setting aggravates coagulopathy by HCA. Thus, we conducted the present study in vivo to clarify whether HCA with a lower temperature at 20 °C worsens coagulopathy in cardiac surgery in comparison to a moderate temperature setting at 28 °C using a pig model.

## Materials and methods

### Animals

This study was approved by the Institutional Animal Care and Use Committee (approval number: 19010). This study was carried out according to the Asahikawa Medical University Animal Experimentation Regulations and the National Research Council’s criteria. Animals were obtained from SANKYO Lab Service Corporation Inc. (Tokyo, Japan) and received care at the Animal Laboratory for Medical Research at Asahikawa Medical University. A total of ten female pigs, with an approximate body weight of 30 kg (30 ± 1.5 kg), were divided at random into the following two groups: moderate temperature at 28 °C (group M, n = 5) and lower temperature at 20 °C (group L, n = 5) of HCA.

### Experimental design

**Fig. 1:**
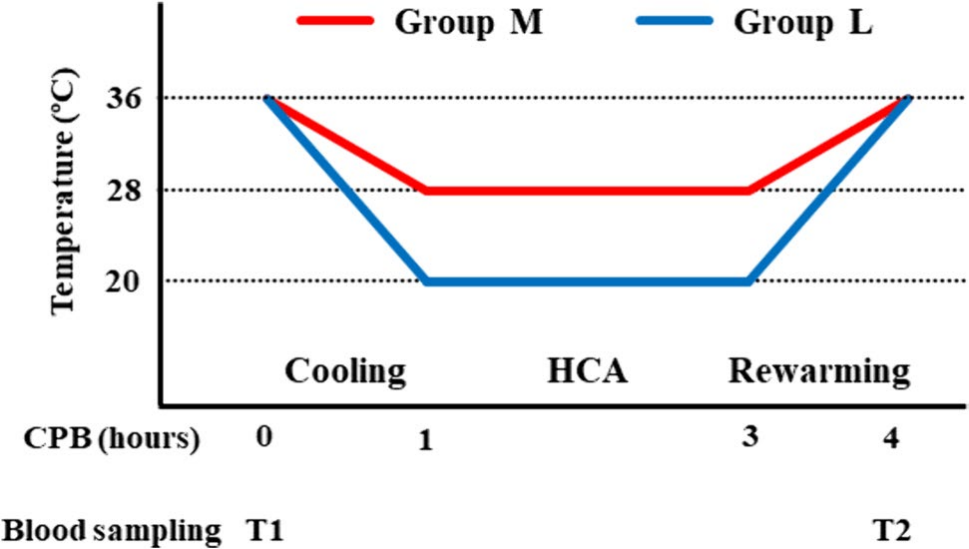
Design of pig cardiopulmonary bypass (CPB) model Pigs were divided into group M (hypothermic circulatory arrest [HCA] at 28 °C, n = 5) and group L (HCA at 20 °C, n = 5). Blood samples were taken at T1 (before operation) and T2 (end of operation). Total CPB operation time was 4 h, including 1 h of cooling, 2 h of HCA, and 1 h of rewarming in both groups

Schematic experimental design of this study is shown in Fig. 1. Pigs were divided into group M and group L. First, blood samples were collected at the beginning of the operation (T1). Cooling was started soon after the beginning of cardiopulmonary bypass (CPB) and took 1 h to reach the targeted rectal temperature (20 °C or 28 °C). HCA with 20 °C or 28 °C was performed for 2 h, and pigs were rewarmed until they reached 36 °C for 1 h. CPB was weaned at the end of rewarming, and blood samples were then collected (T2). Simple HCA, without cerebral perfusion, was chosen since we focused on the influence of hypothermia on coagulation during HCA in the present study. In a clinical setting, HCA is performed for 2 h, at most, or for a shorter period of time. Therefore, we considered that 2 h of HCA was sufficient to investigate the influence of hypothermia on coagulation during HCA. Our previous in vitro study compared coagulation ability after storage of blood at 20 °C and 37 °C [11]. However, in clinical setting of aortic surgery, HCA is performed commonly at 26–28 °C, but not at 37 °C. Therefore, in this in vivo study, we chose clinically standard temperature 28 °C as control to compare with lower temperature 20 °C considering body responses against these temperatures during CPB.

Pigs were assigned into two groups at random in this study; however, basal values of measurements before cooling at T1 differed between group M and group L owing to individual variability. Therefore, the data are normalized and shown as relative values against each group average (100%). The normalization was calculated as follows. The mean value for the measurement of each group (T1 avg) was defined as 100%. Individual data was calculated as a relative value vs. 100 % ([individual T1 measurement/T1 avg] × 100 = T1 [%]; [individual T2 measurement/T1 avg] × 100 = T2 [%]). The normalized T1 (%) and T2 (%) were used for further analysis.

### Operation

Pigs were sedated with an intramuscular injection containing a combination of medetomidine (0.06 mg/kg), midazolam (0.2 mg/kg), and butorphanol (0.2 mg/kg). Then pigs were intubated, placed into mechanical ventilation, and anesthetized with inhalation of 2% isoflurane. Electrocardiogram, oxygen saturation, blood pressure, and rectal temperature were monitored during the operation. An intravenous catheter (Surflo 18G, Terumo, Japan) was placed into the auricular vein for fluid infusion. An arterial sheath (Radifocus introducer II 4Fr, Terumo, Japan) was inserted via the right femoral artery for blood sampling and monitoring blood pressure. Blood samples at T1 were collected soon after the placement of the arterial sheath. Before CPB, 300 IU/kg of heparin was administrated.

The CPB circuit consisted of a roller pump (Stockert S3, LivaNova, UK), reservoir, and oxygenator (CX-FX05RW, Terumo, Tokyo, Japan) with a heater unit (HHC-51, Senko Medical, Tokyo, Japan) and was primed with bicarbonate Ringer’s solution and mannitol. CPB flow was maintained at 70–100 mL/min/kg during complete CPB. A mean blood pressure > 60 mmHg was maintained during surgery, except during HCA.

After the standard median sternotomy, CPB was established by bicaval venous cannulation (18 Fr & 22 Fr, Medtronic, USA). Arterial cannulation was performed at the level of the ascending aorta (16 Fr, Medtronic, USA). A venting tube (10 Fr, Medtronic, USA) was placed into the left ventricle via the apex. After the end of the cooling, the ascending aorta was clamped, and 20 mL/kg of St. Thomas II cardioplegia (Miotector, Mochida Pharmaceutical Co. Ltd, Tokyo, Japan) was administrated via the root of the ascending aorta. Cardioplegia was administrated every hour during HCA. After the end of the HCA, rewarming began. When the pigs were rewarmed to 36 °C, they were weaned from CPB, and protamine was administrated (1:1 ratio to the applied heparin dosage). Blood samples at T2 were collected 5 min after the administration of protamine. Pigs were euthanized by the administration of KCl at the end of the operation.

### Blood sampling

Around 10 mL of blood was obtained from the femoral arterial sheath at each time point and was placed into 2.7-mL citrated vacuum tubes (BD Vacutainer, 0.109 M sodium citrate) for standard laboratory tests (SLTs) and rotational thromboelastometry (ROTEM) and into 2.0-mL ethylenediaminetetraacetic acid (EDTA) vacuum tubes (BD Vacutainer, K2-EDTA 1.8 mg/mL) for SLTs.

SLTs were assayed by using a fully automated hematology analyzer XN-3000 (Sysmex Corporation, Kobe, Japan) and a fully automated coagulation analyzer CS-5100 (Sysmex Corporation, Kobe, Japan). Hemoglobin (Hb), fibrinogen, platelet count (Plt), prothrombin time (PT), and activated partial thromboplastin time (APTT) were analyzed.

ROTEM analyses were performed with ROTEM delta (Tem Systems Inc., Munich, Germany) according to the manufacturer’s instructions. EXTEM and FIBTEM assays were performed. EXTEM assays show clot strength with extrinsic pathway activation of whole blood with tissue factor. FIBTEM assays also indicate clot strength, but platelet contribution to the clot strength is prevented by the addition of cytochalasin D. In ROTEM analyses, clotting time (CT) is the latent time before the start of clot formation, and clot formation time (CFT) is the time from CT until the clot reaches 20 mm in size, indicating the speed of clot formation. The maximum clot firmness (MCF) is the largest vertical amplitude of the trace, reflecting the absolute strength of fibrin and platelet clots. The maximum clot elasticity (MCE) is calculated as follows: (MCF × 100)/(100 − MCF) to accommodate Hook’s law, and it represents the actual physical properties of the clot. We measured MCF and MCE in EXTEM and FIBTEM assays to assess clot strength. CT and CFT in EXTEM were measured to assess the clot formation status.

### Statistical analysis

**Fig. 2:**
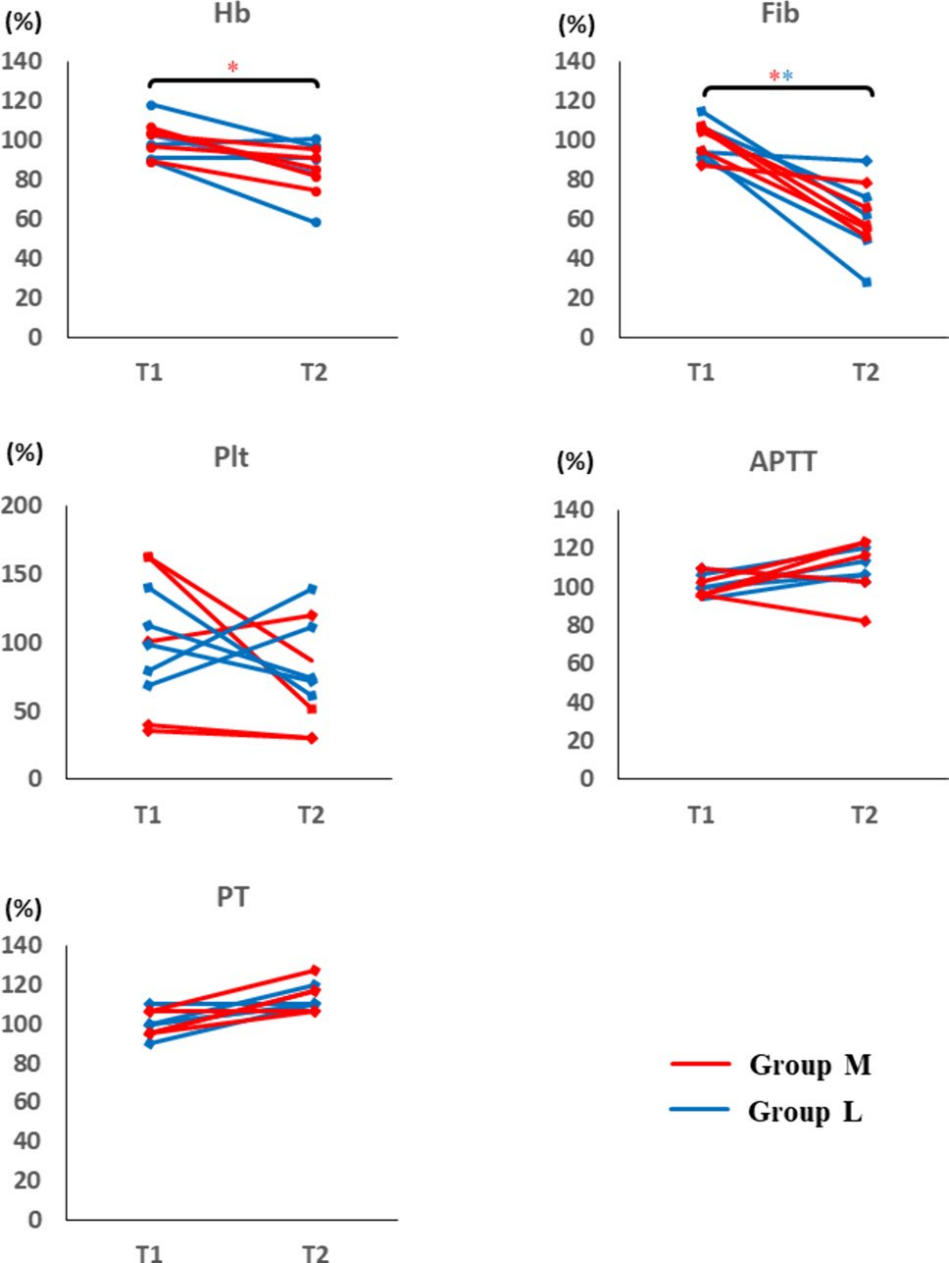
Effect of cardiopulmonary bypass on coagulation in standard laboratory tests (SLTs) Coagulation capability was evaluated by SLTs. The data are shown as relative values against the averaged value at T1 for each group (100%). The data points represent the individual data at T1 and T2. Group M in red and H in blue. * represents p < 0.05 (T2 vs. T1). *Hb* hemoglobin, *Fib* fibrinogen, *Plt* platelet count, *PT* prothrombin time, *APTT* activated partial thromboplastin time

**Fig. 3:**
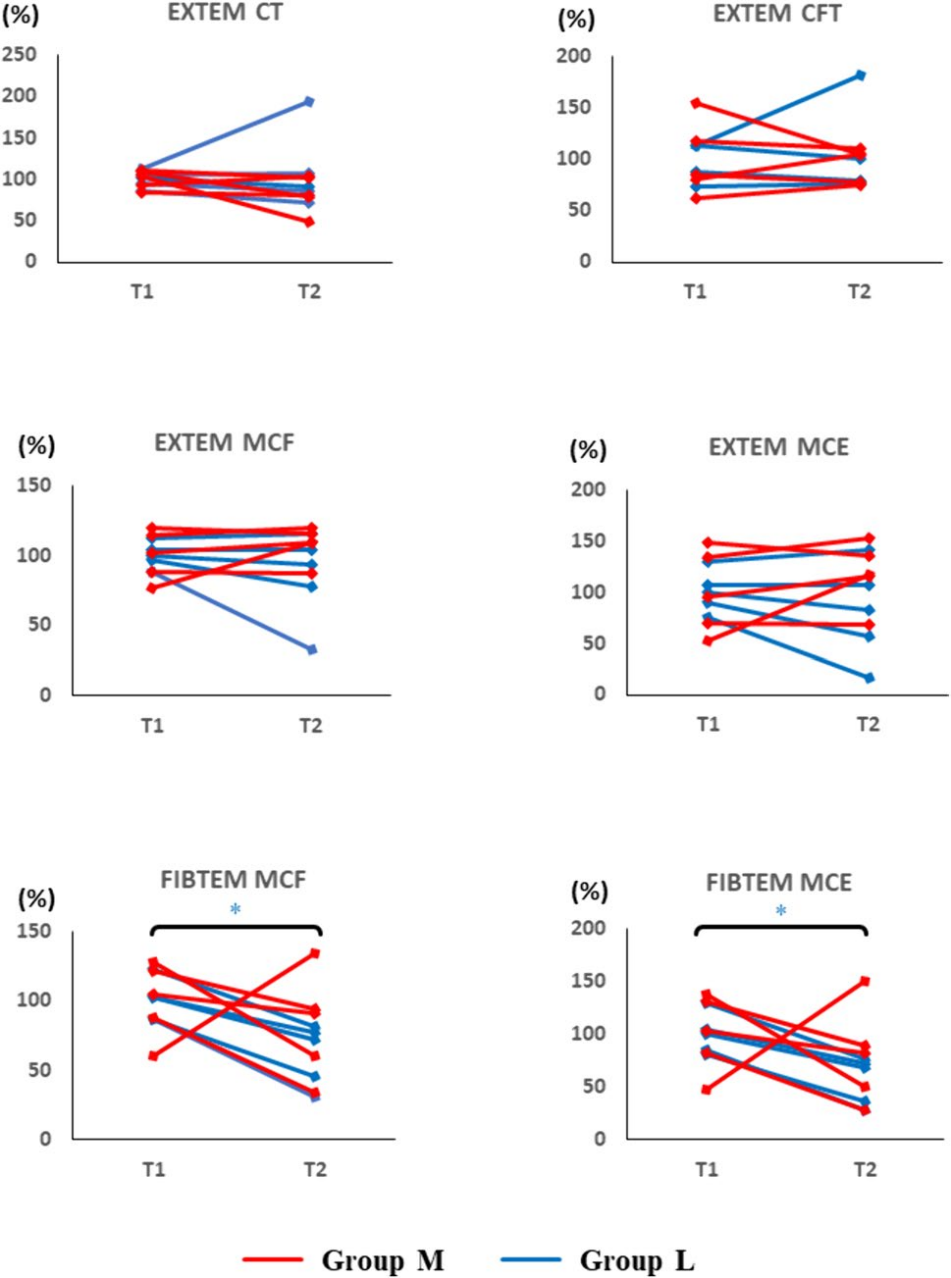
Effect of cardiopulmonary bypass on coagulation in rotational thromboelastometry (ROTEM) Coagulation capability was evaluated by ROTEM. The data are shown as relative values against the averaged value at T1 for each group (100%). The data points represent the individual data at T1 and T2. Group M in red and H in blue. * represents p < 0.05 (T2 vs. T1). *CFT* clot formation time, *MCF* maximum clot firmness, *MCE* maximum clot elasticity

**Fig. 4:**
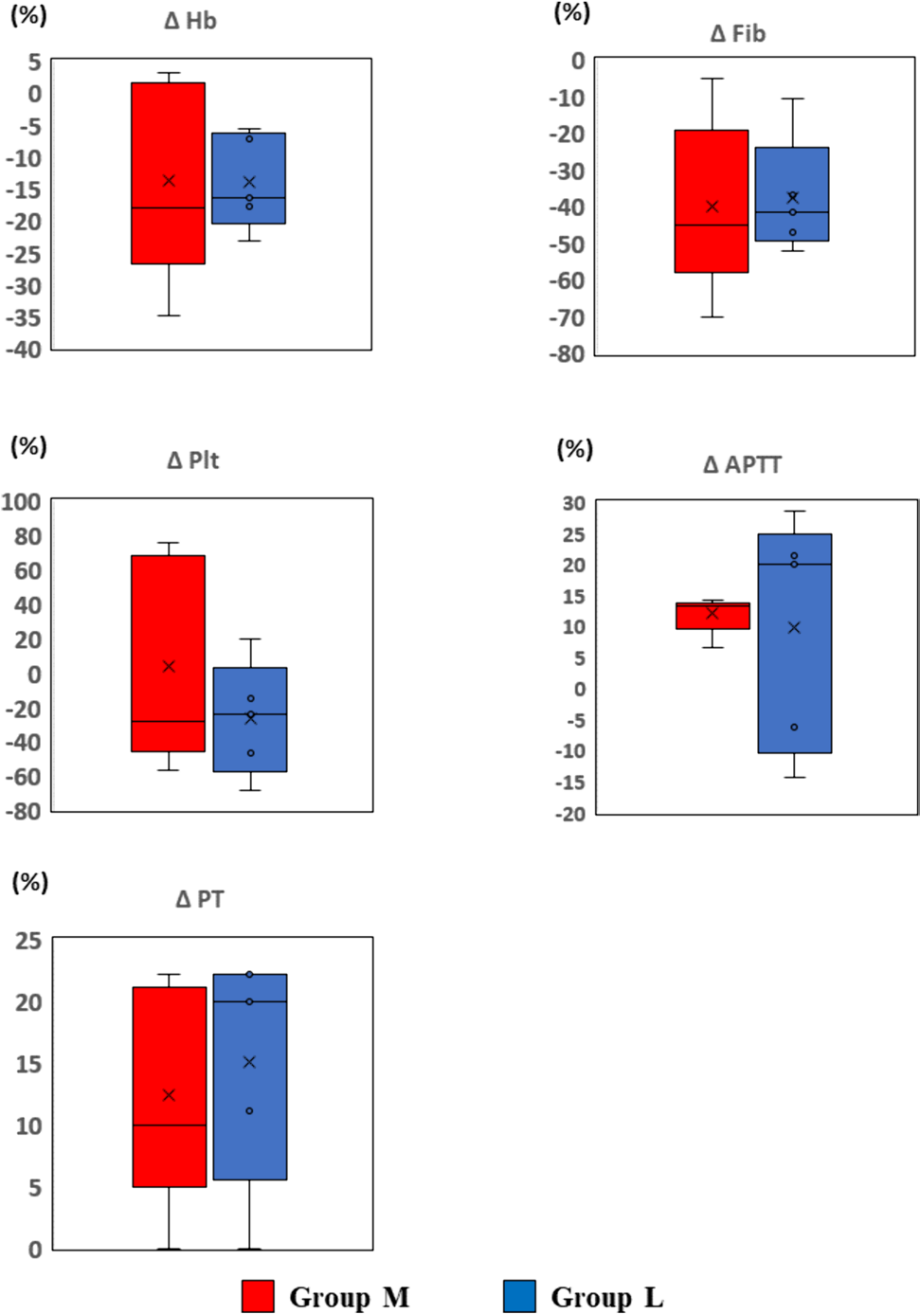
Effect of hypothermic circulatory arrest temperature setting on coagulation in standard laboratory tests (SLTs) Shown are the changes in SLTs parameters after the operation (T1 − T2). Change (%) = ([T2 measured value − T1 measured value]/[T1 measured value]) × 100. *Hb* hemoglobin, *Fib* fibrinogen, *Plt* platelet count, *PT* prothrombin time, *APTT* activated partial thromboplastin time

**Fig. 5:**
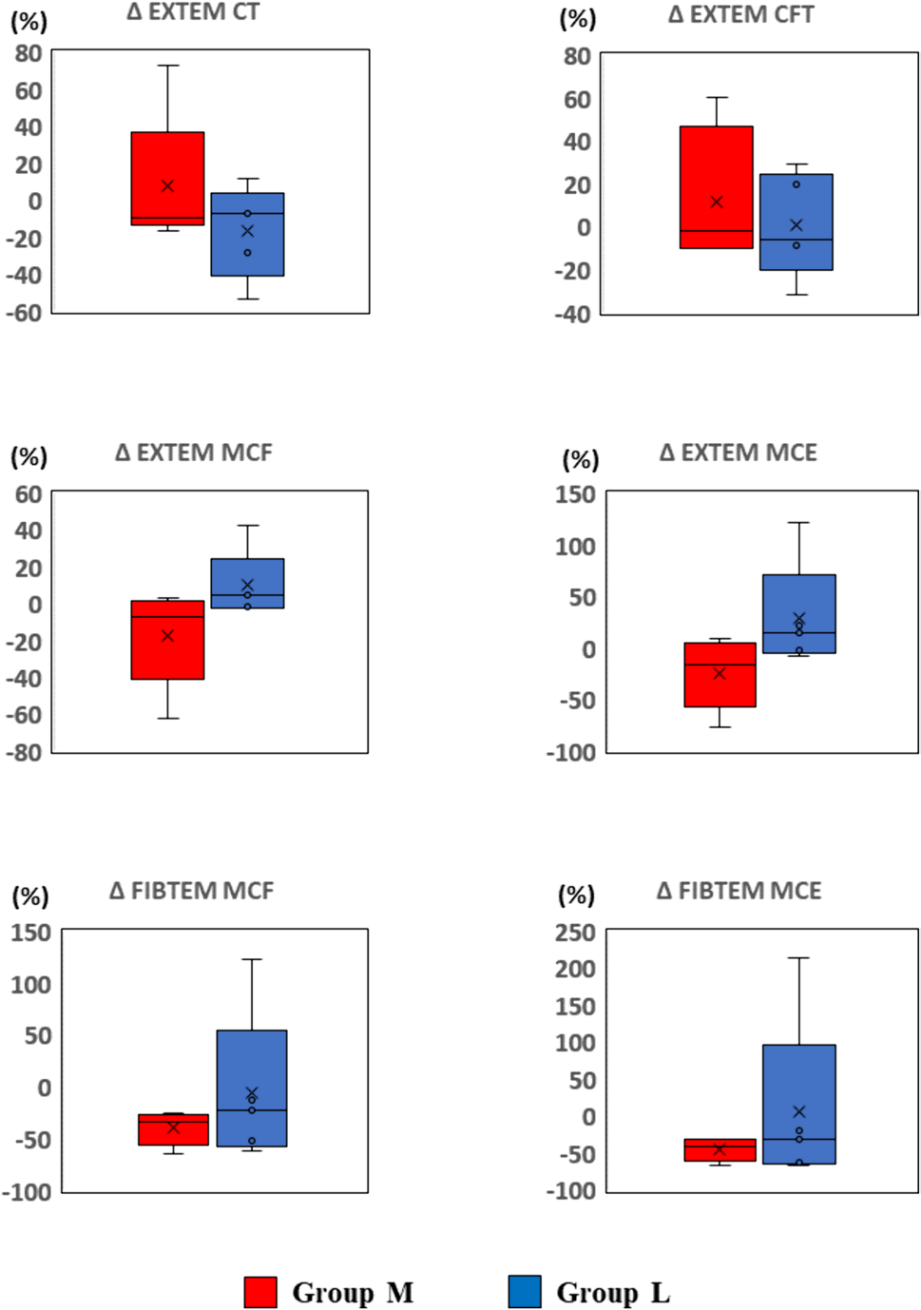
Effect of hypothermic circulatory arrest temperature setting on coagulation in rotational thromboelastometry (ROTEM) Shown are the changes in ROTEM parameters after the operation (T1 − T2). Change (%) = ([T2 measured value − T1 measured value]/[T1 measured value]) × 100. *CFT* clot formation time, *MCF* maximum clot firmness, *MCE* maximum clot elasticity

All statistical data were analyzed by using EZR (Saitama Medical Center, Jichi Medical University, Saitama, Japan), which is a graphical user interface for R (The R Foundation for Statistical Computing, Vienna, Austria) [12]. Values are described as individual values (Figs. 2, 3) or medians with quartiles (Figs. 4, 5). A paired *t*-test was used to compare continuous variables between the two time points (T1 vs. T2) in the same sample of each group. Welch’s *t*-test was used to compare the changes of each parameter between the groups (group M vs. group L). Statistical significance was defined as p < 0.05.

## Results

### Effect of CPB on coagulation (T2 vs. T1)

First, we analyzed the influence of CPB on coagulopathy by comparing SLTs and ROTEM between T1 and T2 in both groups (Figs. 2, 3). Pigs were assigned into two groups at random; however, basal values before cooling at T1 had differences between group M and group L owing to individual variability. Therefore, the data were normalized and shown as relative values against each group average (100%).

In SLTs analyses, Hb in group M (p = 0.016) and fibrinogen in both groups (group L, p = 0.018; group M, p = 0.008) were significantly decreased after CPB. Plt was equivalent at both time points in both groups. APTT and PT had no significant difference between T1 and T2.

In ROTEM analyses, all parameters of EXTEM assays showed no significant differences between T1 and T2. In the FIBTEM assay, both MCF and MCE in group L significantly decreased (MCF, p = 0.001; MCE, p < 0.001). Group M also showed a decreasing tendency of MCF and MCE, but the difference was not statistically significant as one sample did not follow this pattern.

These results indicated that regardless of temperature setting, hypothermic CPB decreased fibrinogen in SLTs and decreased FIBTEM MCF and MCE in ROTEM.

### Effect of lower temperature on coagulation (group L vs. group M)

Next, we compared degrees of changes in SLTs and ROTEM during CPB (T1 to T2) to clarify the influence of a lower temperature setting of HCA on coagulation. The changes in all parameters in SLTs were comparable between the groups (Fig. 4). In ROTEM analysis, CT, CFT, MCF, and MCE of the EXTEM assay were comparable between the groups. The FIBTEM assay also showed no significant difference between the groups. Decreases in MCF and MCE in the EXTEM assay were both within ± 20%, but the FIBTEM assay showed an approximate 50% decrease in both groups (Fig. 5).

These results indicated that a lower temperature setting did not have a significant impact on coagulopathy under HCA in cardiac surgery.

## Discussion

We conducted the present study to clarify whether a lower temperature setting of HCA in aortic surgery worsens coagulopathy in vivo, employing a pig CPB model. Our results revealed two important findings. First, CPB decreased fibrinogen in SLTs and FIBTEM MCF and MCE in ROTEM. Second, a lower temperature setting of HCA did not affect the coagulation ability measured by SLTs and ROTEM, compared with a higher temperature setting. These results confirm the findings of our previous in vitro study and suggest that a lower temperature itself does not deteriorate coagulopathy caused by HCA, at least under the experimental condition without complex manipulation on the aorta.

It has been believed that a lower temperature setting of HCA makes coagulopathy worse in aortic surgery, and many papers report increased bleeding amounts in aortic surgery under HCA [5, 13]. However, there is no clear evidence in a clinical setting that a lower temperature setting of HCA actually worsens coagulopathy because the mechanism of coagulopathy under HCA is complex, making it difficult to clarify causation. To investigate a single effect of the temperature setting in HCA, in vitro and animal studies with the elimination of clinical factors are mandatory, but to the best of our knowledge, such reports are lacking. Therefore, we performed the previous in vitro study to clarify the influence of hypothermia on blood [11]. The results demonstrated that hypothermic temperature itself did not cause irreversible coagulopathy in vitro and suggested that other factors, such as body reaction to hypothermia, might worsen coagulopathy during HCA.

### CPB-induced coagulopathy

It is well known that CPB causes coagulopathy, and longer CPB time results in an increased risk of bleeding. During CPB, various factors, such as activation of platelet, inflammation, or fibrinolysis, cause coagulopathy, resulting in life-threatening bleeding [5, 14]. In this study, we employed ROTEM together with SLTs to evaluate coagulation status since it is reported that ROTEM can precisely analyze the nature of coagulation [15].

Our results, testing the effect of CPB on coagulation, indicated that CPB decreased fibrinogen. Hb also showed a reduced trend and represented blood dilution by the CPB circuit. However, the degree of reduction in fibrinogen is larger than that of Hb (Figs. 2, 4), suggesting additional mechanisms together with dilution (e.g., degradation and loss of production). Consistent with this data, FIBTEM MCF and MCE were reduced, indicating reduced strength of the fibrin polymer in the clot. Considering our data and the fact that fibrinogen is related to fibrin, reduction of fibrinogen is a likely cause of coagulopathy-induced CPB.

Other measurements in SLTs and ROTEM in the present study showed no significant differences between T1 and T2. The findings suggested that FIBTEM could be a sensitive predictor of coagulopathy in CPB. Other previous studies support our present findings, which report that a decrease of FIBTEM MCF in ROTEM or functional fibrinogen in thromboelastography could predict coagulopathy during CPB before PT and APTT showed changes [16, 17]. The European Association of Cardiothoracic Anaesthesiology also reported that monitoring of FIBTEM MCF can detect bleeding during cardiac surgery earlier and more precisely than Fib in SLTs, and it is a useful parameter to determine the administration of fibrinogen concentrate [18].

### HCA-induced coagulopathy

HCA has been considered one of the main factors that causes and worsens coagulopathy during aortic surgery [5, 14]. Previously, deep hypothermic circulatory arrest at < 20 °C was commonly performed in aortic surgery. As the management of cerebral perfusion improved, surgeons have been trying moderate hypothermic circulatory arrest (MHCA at 20–28 °C) or mild HCA (> 28 °C) to reduce CPB time and bleeding [6–8]. We previously reported that bleeding and transfusion of cardiac surgery under HCA were much higher than those of general cardiac surgery [13]. Recently MHCA, at around 26–28 °C with selective cerebral perfusion, became more common, and many studies reported on MHCA without any increase in complications [6, 8, 19]. Furthermore, Watanabe et al. reported that a total arch replacement at 32 °C HCA was performed and reduced approximately 40% of bleeding and 50% of transfusion compared with the MHCA group [20]. Some studies also reported no significant difference in the bleeding and transfusion among different temperature settings in cardiac surgery under HCA [9, 19]. HCA is performed to protect organs and neurological system from ischemic injury. It has been reported that HCA with cerebral perfusion at 28 °C is beneficial for organ and neurological system protection up to 90 min [21], however higher temperature might damage organs. In this study, we demonstrated that HCA at 20 °C for 2 h had no significant impact on coagulation ability after rewarming in pig model. Base on this finding suggesting that a lower temperature setting is not a direct cause of coagulopathy after HCA, recent attempts using a higher temperature setting of HCA may take the risk of organ damage, without preventing coagulopathy [21, 22].

### HCA and fibrinogen

Our results showed no significant effect of a lower temperature setting on fibrinogen, FIBTEM MCF, or FIBTEM MCE induced by HCA.

Whelihan et al. investigated the influence of hypothermia on coagulation ability in vitro in three different temperature groups (37 °C, 32 °C, and 27 °C) [23]. They reported that a lower temperature setting delayed consumption of fibrinogen to form fibrin in vitro, whereas maximum clot strength contributed by fibrinogen was not affected. Our previous in vitro study also showed that clot strength contributed by fibrinogen was not affected under hypothermia (20 °C) with rewarming until the temperature reached 37 °C [11]. These studies demonstrated that hypothermic temperature itself did not affect clot strength contributed by fibrinogen in vitro.

In contrast to this in vitro study, Martini et al. reported that hypothermia caused a significant decrease in fibrinogen synthesis in a pig model [24]. However, our data showed no significant difference in fibrinogen, FIBTEM MCF, or MCE between 20 °C and 28 °C HCA after rewarming. This finding suggested that reduced fibrinogen synthesis could recover enough during rewarming, and the lower temperature was not a disadvantage in fibrinogen-mediated coagulation in vivo when comparing 20 °C and 28 °C of HCA.

### Limitations

Our present study has several limitations. First, we did not perform any manipulation of the aorta in this pig model. In the clinical setting of aortic surgery under HCA, the aorta is incised and anastomosed with a prosthesis. The suture line is one of the most common factors of surgical bleeding. Thus, any additional surgical manipulation of the aorta might result in a different result from our present study. Second, we performed simple HCA without cerebral perfusion for two hours to clarify the influence of hypothermia on coagulation. In reality, simple HCA is not used this long without cerebral perfusion because its risk of cerebral damage. HCA with concomitant cerebral perfusion might result in a different outcome. Third, we assessed coagulation only before and after CPB, but not during HCA. Therefore, our study cannot rule out a potential coagulopathy during hypothermia may be related to the amount of intra-operative bleeding while prolonged CPB, even though our data demonstrated that coagulation ability was recovered after rewarming. Fourth, there is no procedure of “pump-suction” in this study. Pump-suction can damage blood cells and activate the inflammatory reaction, which could cause coagulopathy in the clinical setting of aortic surgery under HCA [25].

Despite these limitations, we believe this study is noteworthy to clarify the mechanism of coagulopathy under HCA.

### Conclusion

Our present study indicated that hypothermic CPB decreased the contribution of fibrinogen to coagulation, however, a lower temperature setting of HCA at 20 °C for 2 h did not significantly affect coagulopathy compared to that of HCA at 28 °C after re-warming to 37 °C. As our previous in vitro study and the present in vivo study showed that a lower temperature setting of HCA did not affect coagulation capability, a higher temperature setting of HCA may not help to reduce the bleeding during aortic surgery. However, further studies in clinical settings will be needed.

## Data Availability

Not applicable.
